# Proceedings of the Genesee County Medical Society at Its Semi-Annual Meeting at LeRoy

**Published:** 1863-07

**Authors:** 


ART. III.—*Proceedings of the Genesee County Medical Society at its Semi-Annual Meeting at LeRoy.*

BATAVIA, June 13, 1863.

The Semi-Annual Meeting of the Genesee County Medical Society was held at Dr. S. Barrett's office, LeRoy, Tuesday, June 9, 1863, Dr. C. M. Smith, Vice President, in the Chair.

The session was mainly devoted to discussion of medical topics and reports of cases. Dr. Barrett, delegate to the State Society, gave a verbal report of various interesting incidents which transpired during the meeting. It was regretted by the Society that the State Transactions had not been received for distribution.

Dr. Root reported two cases of lithotomy—one a female, the other a male, which had occurred in his practice during the last three months.

*Case* 1—Mrs. Wm. Francis, aged 33 years, the mother of four children, had been suffering with symptoms of urinary calculus for about four years. During this time she had borne two children, the youngest at the time of the operation being about five weeks old. The stone was ascertained to be a large one by examination per vaginam and sounding, and hence no effort was made to extract without an incision.

March 5th, assisted by Drs. Miller and Ellenwood, the patient fully under the influence of chloroform, and in the usual position, a straight grooved staff was passed into the urethra, and this canal laid open its whole length with a probe pointed bistoury. The knife being withdrawn the left index finger was passed into the bladder over the staff, and came in contact with the stone. The staff was then removed and a medium sized pair of forceps passed over the finger into the bladder and the stone was seized without difficulty, but in attempting to extract it the calculus was crushed, and I was obliged to remove it piece-meal. The bladder was washed out by injecting warm water, but all the small particles could not be easily removed while the patient was in the recumbent position; some were left with the expectation that they would come away while urinating on the vessel; next day this happened. There was some fever for two or three days following the operation which gradually subsided, and the patient made a good recovery. There has been slight incontinence of urine when she has been much on her feet, but this has now wholly disappeared. The *calculus* was about the size of a hen's egg—the outer part hard, forming a kind of shell; the nucleus, which was about an inch in diameter, also hard, and removed entire. The chemical composition of all, except the nucleus, was the triple phosphate of ammonia and magnesia. The nucleus was composed of phosphates of lime.

*Case* 2.—George Memsted, aged 30 years, American, has suffered from symptoms of urinary calculus for the last twelve years. He would occasionally have "attacks of stone," and afterwards be in comparatively good health, but never could ride horseback or in a wagon over a rough road. About two weeks before the operation (May 21st) he came under observation, and the presence of stone ascertained; but his symptoms were so bad that it was not thought prudent then to interpose, but to wait with the hope that he might improve. He suffered from chills and fever and profuse perspiration, scalding urine, which deposited large quantities of thick mucus and pus, urine alkaline, loss of appetite and great emaciation. Notwithstanding the means which were used with the council of Dr. Cotes of this place, the symptoms did not improve, the chills recurred with great regularity; the urine was copious and alkaline; the pulse at no time was much less than 120. Without waiting longer it was decided that the only chance for recovery was to perform the operation immediately.

May 21st, assisted by Drs. Cotes, Pampilon and Warren, the lateral operation was performed, and the stone extracted. The patient was under the influence of chloroform administered by Dr. Warren. No arteries were divided and the hemorrhage was slight. There was no difficulty in seizing the stone, which was large, measuring six inches in its greatest circumference and five in its smallest, weighing nearly three ounces. A silver tube was introduced into the bladder and secured by tapes to the patient's waist. There was no exhaustion apparent as resulting from the operation. The patient was put to bed and a full anodyne administered. I visited him in five hours; secondary hemorrhage had come on without my being notified, but had ceased by clots forming around the silver tube. There was some prostration, resulting from the loss of blood, but he rallied well. On the third day the tube was removed. By the fifth day the wound was partially closed, and he began to have desire for his urine to pass off by the urethra. After this, for some cause not apparent, he began to lose strength; the wound ceased to heal; no food could be borne on the stomach, and he died ten days after the operation. No post mortem was allowed. No doubt there was disorganization of the bladder which was the cause of the chills and other constitutional symptoms. The chills however did not recur after the removal of the calculus. The chemical composition of the calculus was uric or lithic acid. Its internal structure was lamellated. Its external surface finely tuberculated and of a dark mahogany color. Figures 436 and 437, Gross' Surgery, finely represents the external surface and internal structure of this calculus.

Dr. Barrett reported the following cases:

*A Case of Acute Peritonitis, treated successfully by large doses of sulphate morphine.*

January 2, 1863, I was called to see Miss J. C., aged 12 years. She had been sick two days. I found her with pulse 100 per minute; furred tongue, irritable stomach; bowels tympanitic, and excessively tender over the lower portion; the slightest touch gave her acute pain; heat of skin and great thirst. She had taken a cathartic which distressed her very much, and left her bowels in an irritable state, with tenesmus; soreness increasing; urine scanty and high colored and pain in voiding. I ordered fomentations of hops to the bowels, and mustard poultices if not relieved; gave Cal. 2 grs., Dover's Powder 3 grs. every 4 hours.

3d. The tenesmus is relieved, but pain and soreness no better; continue treatment; if bowels are not relieved give ℥ss. Cast. Oil in the morning.

4th. Oil given and bowels evacuated pretty freely; symptoms about the same, except becoming more restless and the soreness extending higher; continue Dover's Powder and fomentations.

5th. Had a very restless night; abdomen very tense and exceeding tender; soreness extending to epigastrium; great pain in making effort to void urine, with constant desire to do so; pulse 120; gave her 1 gr. sulph. morphine, and directed the dose to be repeated every 3 hours.

6th. The first dose of morphine relieved her very much; has had a very comfortable night; soreness greatly diminished; has urinated well, and the bowels have moved freely and easily; pulse 90, and soft; continue morphine.

7th, Still improving; no pain, and but very little soreness; skin soft and moist; sleep has been refreshing; pulse normal; morphine gr. ss every 6 hours.

9th, Convalescing rapidly; sat up some to-day; continue treatment.

11th. Still improving; she continued to convalesce with good appetite, until the 16th, when she over-ate, which produced vomiting and some soreness over the abdomen, with ardor urinæ. The morphine was again given every 3 hours, with prompt relief. She now convalesced rapidly, and in one week returned to Buffalo where she had been attending school.

*A Case of Fragilitas Ossium.*—Miss M. M., aged 40, has been in poor health several years; had a tumor in the left breast, which was removed by Dr. Parker of New York, some two years since; she has since been under hydropathic and other irregular treatment. For the past year and a half she has been mostly confined to her bed, suffering great pain through the back and hips, especially on making any attempt to change her position. The 15th of February last I was called to see her in consultation with Dr. Wells, of Caledonia, who was then in attendance; about two weeks previous in attempting to move or turn herself, she felt a sharp pain in the left hip joint; the toes and knee gradually turned inward, and the trochanter major projected backward upon the dorsum of the illium. Owing to the great nervous irritability of the system, I could not make a satisfactory examination, and could only prescribe palliatives, feeling a doubt whether there was a fracture or not.

The 6th of April I was again called to see her, and found that a few hours before in attempting to raise her right leg with her hands, the femur broke instantly with loud snap. There was a transverse fracture at the junction of the middle and upper third. We placed the limb upon a double inclined plane, with the usual dressings. She had had slight pain and swelling of the periosteum at the point where the fracture took place for some days previous. At the expiration of eight weeks I removed the dressing; got her consent to take chloroform and be placed upon a stretcher; in doing which I brought the other limb into position, and found there was a fracture of the neck of the femur; crepitus was distinct; the right femur there was some provisional callus thrown out around the seat of fracture, but not enough to hold the fragments in coaptation; the appetite for the most part has been good. She eats beef-steak with the sparkling wine. She has some periosteal pains of the upper extremities. She is very much emaciated. The menstrual periods have ceased to recur the past year. She has some vaginal discharge, but no organic disease that I can detect. The presumption with me has been, that the tumor removed by Dr. Parker was cancerous. I am confident there is no syphilitic taint in the system, but most probable a cancerous cachexia, so affecting the system as to prevent the proper assimilation of animal matter to make good bone.

Dr. Barrett invited the Society to a bountiful and luxurious dinner.

Adjourned to the Annual Meeting in Batavia, January, 1864.

